# Cord Care Practices: A Perspective of Contemporary African Setting

**DOI:** 10.3389/fpubh.2018.00010

**Published:** 2018-01-31

**Authors:** Tolulope O. Afolaranmi, Zuwaira I. Hassan, Ofakunrin O. Akinyemi, Sarah S. Sule, Matthew U. Malete, Choji Pam Choji, Danjuma A. Bello

**Affiliations:** ^1^Department of Community Medicine, University of Jos, Jos, Nigeria; ^2^Department of Community Medicine, Jos University Teaching Hospital, Jos, Nigeria; ^3^Department of Paediatrics, University of Jos, Jos, Nigeria; ^4^Department of Paediatrics, Jos University Teaching Hospital, Jos, Nigeria; ^5^Faculty of Medical Sciences, University of Jos, Jos, Nigeria

**Keywords:** knowledge, practice, predictors, cord care, African setting

## Abstract

**Background:**

Cord care is the series of steps applied in handling of the umbilical cord after delivery of the new born. Globally, an estimated 4 million deaths occur annually within the first 4 weeks of life and 1.5 million of these deaths are attributable to infections. In Nigeria, studies have reported umbilical cord infections accounting for between 10 and 19% of neonatal admissions and resultant estimated 30–49% neonatal deaths. Hence, this study was conducted to assess the knowledge and practice of cord care within a contemporary setting.

**Methodology:**

This was a cross-sectional study conducted among 324 mothers of children less than 59 months using a multistage sampling technique and SSPS version 20 was used for data analysis. Crude and adjusted odds ratios as well as 95% confidence interval were used in this study with a *P*-value of ≤0.05 considered statistically significant.

**Results:**

The mean age of the mothers in the study was 27.5 ± 6 years with majority of them having good overall knowledge and practice of cord care. Factors such residence in rural community (AOR = 0.26; 95% CI = 0.0915–0.7230) and heath facility delivery (AOR = 7.0; 95% CI = 4.7877–9.3948) were predictors of cord care practices.

**Conclusion:**

This study has brought to light the level of cord care practices with health facility delivery, place of residence, and knowledge of cord care as its determinants.

## Introduction

Cord care is the series of steps applied in handling of the umbilical cord after delivery of the new born and if not meticulously carried out will contributes significantly to newborns’ risk of infection and mortality (1–3). Studies conducted in Cameroon and Nigeria both in the West African sub region reported unsatisfactory levels of cord care practices among mothers bring to bear its importance to increased risk of infections and mortality in the neonatal period (4, 5). World Health Organization advocates for dry umbilical cord care and application of topical antiseptics in situations where hygienic conditions are poor or infection rates are high (3, 6). However the Nigerian government recommends the use of Methylated spirit or chlorhexidine solution for cord care (7, 8). Globally, about 130 million babies are delivered annually with an estimated 4 million deaths occurring within the first 4 weeks of life and 1.5 million of these deaths are attributable to infections (7, 9–11). In Nigeria, several hospital-based studies have reported cases of umbilical cord infections accounting for between 10 and 19% of neonatal admissions resulting in as much as 30–49% of neonatal deaths (12). In view of this huge contributions of cord infections to neonatal morbidity and mortality as well as its associated economic and emotional strains on the mothers and the families at large, that this study was conducted to assess the knowledge and practice of cord care within the context of the community with the aim of shedding more light on the drivers of cord care practice in contemporary African settings.

## Materials and Methods

### Study Area

This study was conducted in two communities of Plateau State, North central Nigeria. These communities were Zarrazong and Angwan Rukuba. Zarrazong, a rural community located in Fobur ward of Jos East Local Government Area (LGA) with an estimated land area of 1,020 km^2^ (13). Angwan Rukuba is an urban community located in Naraguta B ward of Jos North LGA of Plateau State. This is a rapidly developing multi-ethnic urban community within Jos metropolis with an estimated population of approximately 5,000 (13, 14).

### Study Design

This was a cross-sectional study design used to determine practices of umbilical cord care among mothers with children below 59 months in order to allow assessment of knowledge and practice of cord care as it exists in the natural communal setting conducted between March and May 2017.

### Study Population

This comprised of mothers with children below 59 months in households in these two communities.

### Sample Size Determination

The sample size for this study was determined using the appropriate sample size determination formula for a cross-sectional study (15). Where *n* is the minimum sample size, *Z* is the standard normal deviate at 95% confidence interval (1.96), *q* is the complementary probability (1 − *p*), *d* is the precision of the study set at 0.05, and *p* is the proportion of mothers with beneficial practice of cord care of 20.5% (0.205) from a previous similar study (16). This gave a minimum sample size of 263 after addition of 5% to cater for non, poor, and incomplete responses.

### Criteria for Inclusion in the Study

Mothers aged between 15 and 45 years permanently residing in these communities who were willing to participate in the study and had at least one child below 59 months during the study period.

### Sample Technique

A multistage approach to sampling was used in this study; Jos North and Jos East LGAs were selected out of the 17 LGAs in the state using simple random sampling technique by balloting. Following which, Naraguta B and Fobur wards were selected from the list of 20 and 18 wards in Jos North and Jos East LGAs, respectively, using simple random sampling by balloting. Thereafter, simple random sampling technique by balloting was used to select Angwan Rukuba and Zarrazong communities from the list of 28 and 18 communities in Naraguta B and Fobur wards, respectively. All inhabited buildings were listed, and household enumeration was done giving a total of 432 and 230 households (defined as a group of person living together under the same roof and eating from the same pot) in Angwan Rukuba and Zarrazong communities, respectively. All households in the two communities were visited and one eligible mother with at least one child under 59 months was sampled. In polygamous households with more than one eligible woman, simple random sample technique by balloting was used to select one of them.

### Data Collection

A semi-structured interviewer administered questionnaire was used in this study comprising of the following sections; sociodemographic characteristics, knowledge of core care, and cord care practices. The data collection instrument was pretested among mothers with children below 59 months in another LGA prior to the commencement of the study following which it was discovered that majority of the mothers in this African setting had more than one under five children and, therefore, the index child was used as the basis for obtaining information on cord care practices in order to minimize bias from information recall. Three research assistants were trained on the content and method of administration of questionnaire prior to the commencement of the study by the principal researcher. Ethical clearance was sought and obtained from Jos University Teaching Hospital institutional health research ethical committee. Written and verbal informed consents were obtained from all the respondents with confidentiality and anonymity of their responses assured and maintained.

### Scoring and Grading of Responses

The knowledge of cord care was assessed using seven stem questions on knowledge of cord care and two (2) points were assigned to every correct response and 0 to any incorrect response giving a maximum attainable score 14 points. A percentile graph was plotted and scores above the 50th percentile was adjudged as good knowledge and those below the 50th percentile as poor knowledge of cord care.

Cord care was assessed as standard if the cord stump was tied with cord clamp, cut with a clean sterile object, and cleaned with methylated spirit only with no application of any other materials (9).

Practice of cord care was adjudged as good following eliciting all of these responses to the composite cord care practice questions:

– Use of cord clamp for tying the cord after delivery
– Use of sterile scissor/blade for separating the baby from the mother after delivery
– Use of methylated spirit for cleaning the cord
– Non application of any other substance (s) on the cord after cleaning with methylated spirit,
– Hand washing before and after cord cleaning
– Cleaning of cord base before the stump
– Frequency of cord cleaning of three or more times in a day.

### Method of Data Analysis

The data obtained were processed and analyzed using SPSS version 20 where sociodemographic characteristics of the mothers were expressed in frequency and percentage. Mean ± SD was used as summary indices for mother’s age and knowledge scores. Crude and adjusted odds ratios were used as point estimates in the logistic regression model while 95% confidence interval was used as the interval estimate. A probability value of less than 0.05 was considered statistically significant in this study.

## Results

The mean age of the mothers in the study was 27.5 ± 6 years with majority (75.6%) being 30 years and below. Since the study was conducted among mothers, 290 (89.5%) were married; however, 21 (6.5%) were single mothers. The highest educational attainments of the respondents cut across all levels with those with non-formal education numbering 112 (35.0%) and tertiary level of education accounting for 67 (20.6%) of the mothers. The family type of the respondents were assessed as monogamous in 287 (88.6%) and polygamous in 37 (11.4%). Most (84.4%) of the mothers had a maximum of four children with 71.0% of their last children delivered in the health facility. See Table 1.

**Table 1:** Sociodemographic characteristics of the respondents.

Variable	Frequency (*n* = 324)	Percentage
**Age group (years)**		
≤30	245	75.6
30 and above	79	24.1
Mean age(±SD)	27.5 ± 6.0 years	

**Marital status**		
Single	21	6.5
Married	290	89.5
Others^a^	13	4.0

**Highest educational attainment of mother**		
None	112	35.0
Primary	91	28.4
Secondary	54	16.9
Tertiary	67	20.6

**Family type**		
Monogamous	287	88.6
Polygamous	37	11.4

**Number of children**		
≤4	272	84.4
5 and more	52	16.0

**Place of delivery of the last child**		
Health facility	230	71.0
Non-health facility	94	29.0

*^a^Separated, divorced, and widowed*.

*Non health facility: home, prayer houses, and traditional birth homes*.

More than half (61.7%) of the respondents were aware of the concept of standard cord care with a relatively good number of these mothers, 185 (57.1%) affirming that tying, cutting, and cleaning the cord stump with methylated spirit were steps involved in good cord care practices. A few (13.6%) of the mothers stated that allowing the cord stump to dry on its own was good practice of cord care. Majority (73.2%) of the studied respondent mentioned methylated spirit as the most important material used for cord care while hot water as well as salt solution accounting for 179 (55.3%) and 35 (10.8%) of the responses, respectively. Quick cord separation and prevention of infection among others were the benefits of good cord care known by 91 (28.5%) and 198 (61.1%) of the mothers, respectively. The overall level of knowledge of cord care was adjudged as good among 239 (73.8%) with the corresponding 85 (26.2%) being poor level of knowledge, while the mean knowledge score of 9.4 ± 3.7 out of a total of 14 was obtained. See Table 2.

**Table 2:** Knowledge of cord care among the respondents.

Level of knowledge	Frequency (*n* = 324)	Percentage
**Awareness of standard cord care**		
Yes	200	61.7
No	134	38.3

**Awareness of components of standard core**		
Use of herbs on cord only	8	2.7
Keep baby away from visitors	22	6.8
Tying, cutting, and cleaning with methylated spirit	185	57.1
Cord allowed to dry on its own	44	13.6
Others^a^	5	1.5

**Awareness of substance used for cord care^c^**		
Salt solution	35	10.8
Methylated spirit	237	73.2
Hot water	179	55.3
Herbal preparation	18	5.6
Do not know	3	0.9
Others^b^	22	6.8

**Awareness of benefits of cord care**		
Quick cord separation	91	28.1
To prevent infection	195	60.2
To prevent abdominal pain	35	10.8

**Level of knowledge of cord care**		
Poor	85	26.2
Good	239	73.8
Mean knowledge score (±SD)	9.4 ± 3.7 out of 14	

*^a^Other represents toothpaste and petroleum jelly*.

*^b^Others shea butter, petroleum jelly, toothpaste, ash, red sand, native chalk, etc*.

*^c^Multiple responses allowed*.

Following the delivery of babies, the umbilical stump cord is always cut and secured with various materials as it was revealed in this study that 273 (73.1%) of the children born to these mothers had their umbilical cord stump secured appropriately with cord clamps. Furthermore, strings of clothing was used in tying the umbilical cord in 36 (11.1%) of the deliveries as reported by the mothers while tailor’s thread was used for 51 (15.7%) of the babies at delivery. Majority (73.2%) of the mothers affirmed that methylated spirit was used to clean the umbilical cord stump; however, 81 (25.0%) of the mothers used salt solution with saliva, 203 (62.7%) used hot water and herbal preparation was applied by 13 (4.0%). Some of these items were either used alone or in combination with other items. Hand hygiene is an important measure in infection prevention and vital to umbilical cord care. In this study, 130 (40.1%) of the mothers recalled that their hand were washed with water only prior to handling the baby’s cord, 153 (47.3%) used soap and water, and 26 (8.0%) used their clothing to wipe the hands before caring for the umbilical cord stump, respectively. Techniques and approach to clean the umbilical cord is important in achieving a desired outcome as majority (73.2%) of the mother mentioned cleaning the cord base before the stump as the most appropriate method, 64 (19.8%) mentioned cleaning of the cord stump alone, and 13 (4.0%) mentioned cleaning the cord surrounding skin only as the most appropriate method. Assessment of the frequency of umbilical cord care revealed that only 27 (8.3%) of the mothers cleaned the cord and stump more than three times a day, 48 (14.8%) after every diaper change, 197 (60.8%) three times a day, and 43 (13.3%) did the cleaning once a day. Overall assessment of the practice of umbilical cord care was adjudged as good among 252 (77.8%) of the respondents. See Table 3.

**Table 3:** Level of practice of cord care among the respondents.

Characteristics	Frequency (*n* = 324)	Percentage
**Material used in tying cord after delivery**		
Cord clamp	237	73.1
String of cloth	36	11.1
Tailor’s thread	51	15.7

**Substance (s) used for cord cleaning^a^**		
Salt solution	81	25.0
Hot water	203	62.7
Herbal preparation	13	4.0
Methylated spirit	237	73.2

**Substance (s) applied after cord cleaning^a^**		
Menthol ointment	63	19.4
Breast milk	12	3.7
Palm oil	26	8.0
Native chalk/ash	8	2.4
Shea butter oil	35	10.8
Others^b^	9	2.8
Nothing	252	77.8

**Care of the hands during cord care**		
Wash hands with water	130	40.1
Wash hands with soap and water	153	47.3
Clean hands on cloth/wrapper	26	8.0
Clean hands with clean handkerchief	15	4.6

**Method of cord cleaning**		
Clean cord base before stump	237	73.2
Clean cord stump only	64	19.8
Clean surrounding skin only	13	4.0
Clean the material used to tie the cord	10	3.1

**Frequency of cord cleaning**		
Morning, afternoon, evening	197	60.8
Once daily	43	13.3
After each nappy is changed	48	14.8
Several times a day	27	8.3

**Level of practice**		
Poor	72	22.2
Good	252	77.8

*^a^Multiple responses elicited*.

*^b^Petroleum jelly, toothpaste, red sand*.

The odds of engaging in good practice of umbilical cord care among the respondents residing in rural community is 0.26 the odds among the respondents residing in urban community (adjusted OR = 0.26; 95% confidence interval = 0.0915–0.7230; *P* = 0.010). Health facility delivery was found to significantly predict good practice of cord care as its odds was seven times the odds among those who delivered outside the health facility (adjusted OR = 7.0; 95% confidence interval = 4.7877–9.3948; *P* < 0.001). Furthermore, the odds of good practice of cord care among the respondents with poor knowledge of cord care was 0.2 times the odds among those with good knowledge. Other factors assessed did not significantly predict good cord care practice. See Table 4.

**Table 4:** Multiple logistic regression of predictors of good practice of cord care.

Factors^a^	COR (95% CI)	AOR (95% CI)	*P*-value
**Age group (years)**			
>30	0.87 (0.4789–1.5866)	1.84 (0.5869–5.7420)	0.297
≤30	1 (–)	1 (–)	–

**Place of residence**			
Rural	0.25 (0.1387–0.4505)	0.26 (0.0915–0.7230)	0.010
Urban	1 (–)	1 (–)	–

**Number of children**			
5 and above	0.42 (0.2218–0.7965)	0.44 (0.1210–1.5943)	0.211
≤4	1 (–)	1 (–)	–

**Place of delivery**			
Health facility	8.8 (3.4948–12.2216)	7.0 (4.7877–9.3948)	<0.001
Non-health facility	1 (–)	1 (–)	–

**Mother’s level of education**			
Primary	2.1 (0.7418–6.1476)	1.3 (0.2537–6.4378)	0.766
Secondary	5.3 (1.9711–14.5060)	1.6 (0.3515–7.3457)	0.541
Tertiary	6.2 (5.6399–16.2966)	1.7 (0.1407–9.6221)	0.687
None	1 (–)	1 (–)	–

**Family type**			
Polygamous	0.48 (0.117–2.0567)	1.1 (0.0772–1.5579)	0.946
Monogamous	1 (–)	1 (–)	–

**Marital status**			
Single	1.7 (0.4956–6.0630)	3.9 (0.3354–4.4504)	0.278
Others	0.7 (0.1939–2.1789)	2.4 (0.3319–7.6120)	0.384
Married	1 (–)	1 (–)	–

**Knowledge of cord care**			
Poor	0.25 (0.1461–0.4456)	0.2 (0.0766–0.5370)	0.001
Good	1 (–)	1 (–)	–

*COR, crude odds ratio; AOR, adjusted odds ratio*.

*^a^P-value for AOR*.

*Non health facility: home, prayer houses, and traditional birth homes*.

*Others: separated, divorced, and widowed*.

## Discussion

Availability of appropriate information on cord care is vital to its practices, in this study, more than half of the mothers were aware of the details of standard cord care. However, another Nigerian study found fewer than half of the mothers with similar result. This variation could probably be as a result of attendance at antenatal care (ANC) in the course of pregnancy, the quality information accessed during ANC and place of delivery (9).

Variation exists in the awareness of use of methylated spirit as the most appropriate substance used for cord care across the divides of Nigeria as a country as observed in this study when compared with other Nigerian studies. As majority of the mothers mentioned the use methylated spirit most appropriate cord cleansing substance in this study conducted in the North central part of the country compared to slightly above half of the respondents with similar knowledge in a study conducted in the southern part of Nigeria (9). However, another study carried out in Nairobi Kenya also corroborated the knowledge of methylated spirit as most appropriate for core care in slight above half of the mothers (17). Others substances expressed by the mothers for cord care in this study and other related studies were hot water, salt and saliva, herbal preparation, shea butter, tooth paste, red sand, ash among others (9, 17). This implies that traditional and socio-cultural belief systems deliver alternate sources of information with significant influence on how orthodox health information is being taken, processed, and eventually utilized.

Mothers in this study displayed a higher level of knowledge of cord care as most of them were adjudged as having good knowledge, which is in synergy with what was obtained in other studies conducted in Nepal and Nigeria where only few mothers lacked knowledge on cord care (18). Contrary findings were obtained from others studies where less than half to below a quarter of the mothers had adequate knowledge of cord care (9, 19, 20). This variation in the level of knowledge of cord care could be attributable to varying educational levels of the respondents, ease of access to health information, place of delivery, cultural affiliations, and place of residence among others.

Proper care of the cord is essential to the survival of the child and one of the ways of ensuring an event-free first few weeks of life. Methylated spirit was the most commonly used cord cleansing substance in this study as practiced by most of the respondents due to its ability to keep the cord stump clean, dry, and promoting quick cord separation. This is further corroborated by the findings of studies conducted in Ghana and Kano North western part of Nigeria (21, 22). Quite surprising is the huge cord care practice gap on using methylated spirit that exists among mothers as other studies found its use in cord care as low as less than a third of the mothers (4, 16, 23). Others substances used on the cord in this study were salt solution, herbal preparation, hot water among others, which had shared similarity with findings of other studies where herbal preparations, traditional substances, shea butter, breast milk, palm oil, ash, etc., were used (3, 4, 22–26). This has brought to light the importance of culture as a driver of practice of cord care and its underestimated subtle contributions to neonatal morbidity and mortality particularly in resource poor countries. Furthermore, a study conducted in Indian revealed that about half of the mothers did not even apply anything on the cord (27).

Fewer than half of the mothers practiced hand washing prior to handling the cord, which is far lower than the majority who practiced hand washing before cord cleaning in studies conducted in Benin city Nigeria and Uganda (2, 16, 24). The practice of hand washing can significantly reduce cord infection and should be encouraged through health education and awareness campaigns targeted at pregnant women and nursing mothers.

Majority of the respondents were found to engage in overall good practices of cord care as evident by the use of methylated spirit solely, cleaning of the cord from the base to the stump, hand washing prior to cord cleaning, and a higher frequency of cleaning, which is similar to the findings of studies from Ethiopia, Nigeria, India (16, 23, 28, 29). However, non satisfactory level of cord care practices was observed in studies conducted in Cameroon, Nigeria, and Nepal (4, 5, 20). The variation in the practice cord care further reiterates that more concerted efforts has to be put in place to ensure safe and beneficial cord handling practices globally in order to bring to the barest minimum morbidity and mortality related to cord infections. This study could, however, not assess the outcome of cord care as it relates to cord separation time and sepsis within the neonatal period owing to the study design used. In view of this limitation, further studies could be conducted to establish this relationship in a cohort of newborn within the neonatal period particularly in the context of African setting.

Delivery in the health facility, rural residence, and poor knowledge of core care had significant influence in predicting cord care practices in this study. However, in other studies, maternal education, social class, place of delivery, mother’s age, urban residence, use of health facility, parity, and family type were found as predictors of cord care (3, 7, 30). These predictors could play significant role in future research aimed at addressing and sharpening cord care practices in contemporary setting by using them as basis for providing evidence based interventions.

## Conclusion

This study has brought to light the level of cord care practice in these contemporary African settings with health facility delivery, place of residence, and knowledge of cord care as its determinants. It is, therefore, opined that this study will provide the foundation for further studies to structuring appropriate interventions at addressing some of the unbeneficial cord care practices.

## Ethics Statement

This study was carried out in accordance with the recommendations of Helsinki declaration and ethical approval was obtained from Jos University Teaching Hospital institutional health research ethical committee.

## Author Contributions

TA, ZH, OA, SS, MM, CC, and DB all participated in the literature review; concept and design of the study; analysis and interpretation of data; drafting and revising the manuscript; and final approval prior to submission for publication. All authors agree to be accountable for the content of the work.

## Conflict of Interest Statement

The authors declare that the research was conducted in the absence of any commercial or financial relationships that could be construed as a potential conflict of interest.
